# HLA-DRα1-mMOG-35-55 treatment of experimental autoimmune encephalomyelitis reduces CNS inflammation, enhances M2 macrophage frequency, and promotes neuroprotection

**DOI:** 10.1186/s12974-015-0342-4

**Published:** 2015-06-24

**Authors:** Gil Benedek, Roberto Meza-Romero, Kelley Jordan, Lucy Keenlyside, Halina Offner, Arthur A. Vandenbark

**Affiliations:** Neuroimmunology Research, VA Portland Health Care System, Portland, OR USA; Tykeson MS Research Laboratory, Department of Neurology UHS-46, Oregon Health & Science University, 3181 SW Sam Jackson Park Rd, Portland, OR USA; Department of Anesthesiology and Perioperative Medicine, Oregon Health & Science University, Portland, OR USA; Department of Molecular Microbiology & Immunology, Oregon Health & Science University, Portland, OR USA

**Keywords:** Experimental autoimmune encephalomyelitis (EAE), Multiple sclerosis (MS), DRα1-mMOG-35-55 therapy, M2 macrophages, Neuroprotection

## Abstract

**Background:**

DRα1-mouse(m)MOG-35-55, a novel construct developed in our laboratory as a simpler and potentially less immunogenic alternative to two-domain class II constructs, was shown previously to target the MIF/CD74 pathway and to reverse clinical and histological signs of experimental autoimmune encephalomyelitis (EAE) in DR*1501-Tg mice in a manner similar to the parent DR2β1-containing construct.

**Methods:**

In order to determine whether DRα1-mMOG-35-55 could treat EAE in major histocompatibility complex (MHC)-mismatched mice and to evaluate the treatment effect on central nervous system (CNS) inflammation, C57BL/6 mice were treated with DRα1-mMOG-35-55. In addition, gene expression profile was analyzed in spinal cords of EAE DR*1501-Tg mice that were treated with DRα1-mMOG-35-55.

**Results:**

We here demonstrate that DRα1-mMOG-35-55 could effectively treat EAE in MHC-mismatched C57BL/6 mice by reducing CNS inflammation, potentially mediated in part through an increased frequency of M2 monocytes in the spinal cord. Microarray analysis of spinal cord tissue from DRα1-mMOG-35-55-treated vs. vehicle control mice with EAE revealed decreased expression of a large number of pro-inflammatory genes including CD74, NLRP3, and IL-1β and increased expression of genes involved in myelin repair (MBP) and neuroregeneration (HUWE1).

**Conclusion:**

These findings indicate that the DRα1-mMOG-35-55 construct retains therapeutic, anti-inflammatory, and neuroprotective activities during treatment of EAE across MHC disparate barriers.

**Electronic supplementary material:**

The online version of this article (doi:10.1186/s12974-015-0342-4) contains supplementary material, which is available to authorized users.

## Background

Multiple sclerosis (MS) is a complex neurological disease with an autoimmune origin involving both genetic and environmental factors in disease pathogenesis [1, 2]. The major genetic effect has been attributed to the major histocompatibility complex (MHC) region on chromosome 6. Within this region, the highly polymorphic human leukocyte antigen (HLA) class II genes, such as HLA-DRB1 and HLA-DQB1, were associated with disease susceptibility [3–6]. Furthermore, several of the MHC genes which are not polymorphic were shown to be involved in disease progression, including the class II invariant chain (CD74) [7].

We previously demonstrated enhanced CD74 cell surface expression on monocytes in mice with experimental autoimmune encephalomyelitis (EAE) [7]. In addition to stabilizing and chaperoning MHC class II molecules to the cell surface, CD74 serves as the primary receptor for binding macrophage migration inhibitory factor (MIF), a pleiotropic pro-inflammatory protein involved in many inflammatory and autoimmune diseases [8–16]. MIF engagement of CD74 leads to the recruitment and activation of CD44 and CXCR2/4 to initiate signaling pathways necessary for MAPK activation, which leads to cell motility and increased survival [17–19]. Mif^−/−^ (MIF-KO) mice exhibit only acute EAE signs but no further progression of clinical disease, correlating with the role of central nervous system (CNS)-infiltrating monocytes in disease progression [20, 13].

Macrophages and microglia play a central role in multiple sclerosis and EAE, which is affected by the polarization state of these cells [21–23]. Classically activated macrophages (M1) govern the CNS during the early stages of these diseases, secreting pro-inflammatory cytokines and activating effector T cells. During the later phase of disease, alternatively activated macrophages (M2) release anti-inflammatory cytokines and promote tissue repair [22, 24–28].

We recently demonstrated a previously unrecognized immunoregulatory interaction in which the HLA-DRα1 polypeptide can inhibit MIF binding to mouse CD74 and downstream signaling, resulting in rapid and potent treatment of ongoing clinical and histological signs of EAE in HLA-DR2-Tg mice [29]. The binding of the DRα1 construct to both human and mouse CD74 and its subsequent blocking of MIF interaction and signaling thus might represent a natural immunoregulatory role for the DRα1 domain in terminating/regulating MIF-dependent inflammation. In addition, we demonstrated that the potency of the DRα1 domain could be enhanced by addition of a peptide extension (MOG-35-55 peptide) that provided secondary structure not present in DRα1 [29]. The invariable DRα1 domain is present in all human subjects and should not be recognized as immunologically foreign. In order to assess whether effective treatment with DRα1 constructs could occur under *histoincompatible* conditions and thus obviate any need to carry out HLA screening prior to injection, we treated EAE in C57BL/6 mice with DRα1-mMOG-35-55 (a complete MHC mismatch) and evaluated disease progression and CNS inflammation.

Herein, we demonstrate that DRα1-mMOG-35-55 reverses EAE clinical signs in C57BL/6 mice, inhibits infiltration of activated monocytes and CD4^+^ T cells into the CNS, and increases the frequency of CD11b^+^ CD206^+^ (M2) monocytes in the spinal cord. Furthermore, microarray analysis of spinal cords of DRα1-mMOG-35-55-treated DR*1501-Tg mice with EAE revealed that the expression of pro-inflammatory genes was dramatically reduced after DRα1-mMOG-35-55 treatment relative to vehicle treatment, while the expression of myelin basic protein (MBP) and other genes that were shown to be involved in remyelination and axonal survival and regeneration was upregulated.

## Materials and methods

### Mice

C57BL/6 mice were purchased from Jackson laboratory. DR*1501-Tg and DR*1502-Tg mice were bred in-house at the Veterinary Medical Unit, VA Portland Health Care System, and used at 8–12 weeks of age. All procedures were approved and performed according to federal, state, and institutional guidelines.

### DRα1-mMOG-35-55 cloning, production, and purification

**Fig. 1:**
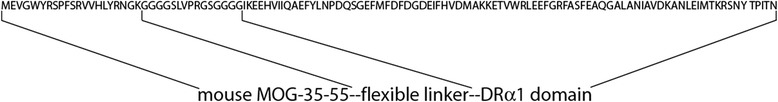
Schematic illustration of the DRα1-mMOG-35-55 construct

Cloning, production, and purification of the DRα1-mMOG-35-55 construct have been described previously [29]. Briefly, DRα1-mMOG-35-55 was built as a single gene joining the mouse (m)MOG-35-55-encoding DNA sequence upstream of the HLA-DRα1 domain with a flexible linker (containing a thrombin cleavage site) between both elements as shown in Fig. 1. This single gene was cloned between the *Nco*I and *Xho*I restriction sites of the pET21d(+) vector, expressed in *Escherichia coli*, and the protein purified following standard purification techniques including anion exchange and size exclusion chromatography in the presence of 6 M urea. Protein was refolded after extensive dialysis in 20 mM Tris, pH 8.5; concentrated; and flash frozen.

### Induction of EAE

DR*1501-Tg and DR*1502-Tg mice were screened for the expression of the HLA marker by flow cytometry [30]. Mice between 8 and 12 weeks of age were immunized s.c. at four sites on the flanks with 0.2 ml of an emulsion of 200 μg immunogenic peptide and complete Freund’s adjuvant containing 400 μg of heat-killed *Mycobacterium tuberculosis* H37RA [30] (Difco, Detroit, MI). In addition, mice were given Pertussis toxin (Ptx) from List Biological Laboratories (Campbell, CA) on days 0 and 2 post-immunization (75 and 200 ng per mouse, respectively). Immunized mice were assessed daily for clinical signs of EAE on a 6-point scale of combined hind limb and forelimb paralysis scores. The following were used for hind limb scores: 0 = no signs; 0.5 = limp tail or mild hind limb weakness (i.e., a mouse cannot resist inversion after a 90° turn of the base of the tail); 1 = limp tail and mild hind limb weakness; 2 = limp tail and moderate hind limb weakness (i.e., an inability of the mouse to rapidly right itself after inversion); 3 = limp tail and moderately severe hind limb weakness (i.e., inability of the mouse to right itself after inversion and clear tilting of hind quarters to either side while walking); 4 = limp tail and severe hind limb weakness (hind feet can move but drag more frequently than face forward); and 5 = limp tail and paraplegia (no movement of hind limbs). Front limb paralysis scores are either 0.5 for clear restriction in normal movement or 1 for complete forelimb paralysis. The combined score is the sum of the hind limb score and the forelimb score. Rarely, there is mortality of mice with severe EAE, and in these cases, mice are scored as a 6 for the remainder of the experiment. Mean EAE scores and standard deviations for mice grouped according to initiation of DRα1-mMOG-35-55 or vehicle treatment were calculated for each day and summed for the entire experiment (cumulative disease index (CDI) represents total disease load).

### DRα1-mMOG-35-55 treatment of EAE

One hundred micrograms of DRα1-mMOG-35-55 protein was injected s.c. daily for 3 or 5 days to treat EAE induced in C57BL/6, DR*1501-Tg, and DR*1502-Tg mice, and clinical signs were scored as described above.

### LPS and MIF in vitro stimulation

Cells were stimulated with 10 ng/ml of lipopolysaccharide (LPS) (*E. coli* 0111:B4) with or without 100 ng/ml recombinant human MIF [31].

### Flow cytometry

Four-color (fluorescein isothiocyanate (FITC), phycoerythrin, (PE), propidium iodide (PI), and allophycocyanin, (APC)) fluorescence flow cytometry analyses were performed to determine the phenotypes of cells following standard antibody staining procedures. For splenocytes, single-cell suspensions of spleens from vehicle- and DRα1-mMOG-35-55-treated groups were prepared by homogenizing the tissue through a fine mesh screen. Cells were pelleted after lysis of red cells followed by washing twice with RPMI. Mononuclear cells from the spinal cord or brain were isolated by Percoll gradient centrifugation as previously described [32]. Cells from the spleen, spinal cord, or brain were resuspended in staining medium (5 % BSA, 1× PBS, and 0.02 % sodium azide) for FACS staining. For intracellular staining, cells were resuspended (2 × 10^6^ cells/ml) in stimulation media (RPMI 1640 media containing 2 % FCS, 1 mM pyruvate, 200 μg/ml penicillin, 200 U/ml streptomycin, 4 mM l-glutamine, and 5 × 10^−5^ M 2-β-ME with phorbol 12-myristate 13-acetate (PMA) [50 ng/ml], ionomycin [500 ng/ml], and Brefeldin A [10 μg/ml] [all reagents from BD Biosciences]) for 4 h. Fc receptors were blocked with mouse Fc receptor-specific mAb (2.3G2; BD Pharmingen) before cell surface staining and then fixed and permeabilized using a Cytofix/Cytoperm kit (BD Biosciences) according to the manufacturer’s instructions. Permeabilized cells were washed with Permeabilization Buffer (BD Biosciences) and stained with either PE-conjugated IFN-γ, APC-conjugated IL-17, or isotype-matched mAb that served as a negative control. Data were collected with CELLQUEST (BD Biosciences, San Jose, CA) and FCS Express (De Novo Software, Los Angeles, CA) software on a FACSCalibur (BD Biosciences) or with Accuri C6 (BD Biosciences).

After staining, cells were washed with staining medium and analyzed immediately with a FACSCalibur using FCS Express (Los Angeles, CA) software or with Accuri C6 (BD Biosciences). Absolute numbers of cells were calculated from live-gated cells. All antibodies were purchased from BD Pharmingen (San Diego, CA), eBioscience (San Diego, CA), or Santa Cruz Biotechnology, Inc. (Santa Cruz, CA) unless otherwise indicated.

### Microarray analysis

Labeled target cDNA was prepared from spinal cord total RNA samples that were pooled from three vehicle-treated or three DRα1-mMOG-35-55-treated DR*1501-Tg mice with EAE. Samples were amplified and labeled according to the Affymetrix GeneChip WT Plus protocol. Amplified and labeled cDNA target samples were each hybridized to Mouse Gene 2.0 ST array. Image processing was performed using Affymetrix Command Console (AGCC) v.3.1.1 software, and expression analysis was performed using Affymetrix Expression Console v.1.1 software.

### Accession code

The accession code is Geo: microarray data GSE68805.

### Functional annotation of expression modules

Gene ontology (GO) category enrichment analysis was performed using WebGestalt software [33, 34]. Briefly, GO enrichment is calculated by comparing the frequency of genes from each different GO present in the set of differently expressed genes to the frequency of genes for the same GO in the mouse genome background.

### Real-time PCR

Whole spinal cords or mononuclear cells that were isolated from spinal cords were isolated from DR*1501-Tg mice. Total RNA was isolated from cells using an RNeasy cultured cell kit according to the manufacturer’s instructions (Qiagen, Valencia, CA, USA). Quantitative real-time PCR was performed using the StepOne Plus system with gene-on-demand assay products (Applied Biosystems) for TaqMan array mouse immune response or for CD74 (Assay ID: Mm00658576_m1), IL-1b (Assay ID: Mm00424228_m1), NLRP3 (Assay ID: Mm00840904_m1), HDAC5 (Assay ID: Mm0124076_m1), HUWE1 (Assay ID: Mm00615533_m1), and MBP (Assay ID: Mm01266402_m1). GAPDH housekeeping gene was amplified as an endogenous control. Primers were used according to the manufacturer’s instructions.

### Statistical analysis

Daily mean scores were statistically analyzed by a two-tailed Mann–Whitney *U* test for a nonparametric comparison between vehicle and DRα1-mMOG-35-55 treatment groups. Mean CDIs were analyzed by Student’s *t* test. Values of *p* < 0.05 were considered significant.

## Results

### DRα1-mMOG-35-55 treatment reverses clinical signs of EAE

**Fig. 2:**
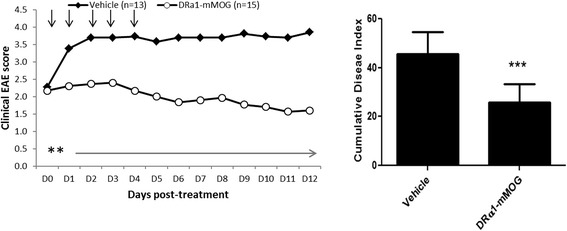
DRα1-mMOG-35-55 treats clinical EAE. C57BL/6 male WT mice with mMOG-35-55-induced EAE were treated after disease onset at a clinical score of 2 with vehicle or DRα1-mMOG-35-55 (100 μg daily × 5, *black arrows*). Mean clinical EAE daily disease scores (*left*) and cumulative disease index scores (*right*) are shown. **p < 0.01, ***p < 0.001. Daily mean scores were analyzed by Mann–Whitney *U* and mean CDI by Student’s *t* test

We demonstrated recently that DRα1-mMOG-35-55 treatment could reverse clinical signs of EAE in DR*1501-Tg mice [29]. In order to test if the DRα1-mMOG-35-55 construct could treat EAE in mice that express the I-A/I-E genes, as opposed to the human HLA-DRα1 and DR2β1 domains in the DR*1501-Tg mice, C57BL/6 mice were immunized with mouse (m)MOG-35-55/CFA/Ptx. Treatment with DRα1-mMOG-35-55 (100 μg daily × 5) after disease onset at a clinical score of 2 significantly reduced clinical EAE scores compared with vehicle-treated mice (Fig. 2).

We further evaluated DRα1-mMOG-35-55 treatment efficacy vs. EAE induced in DR*1502-Tg mice that had minor disparities in both MHC (I-Eα^k^:DR2*1502β chains) and encephalitogenic peptide (hMOG-35-55 peptide containing the S42P substitution). As observed for the C57BL/6 mice, treatment with DRα1-mMOG-35-55 (100 μg daily × 5) after disease onset at a clinical score of 2 significantly reduced clinical EAE scores compared with vehicle-treated mice (Additional file 1: Figure S1). These results indicate that DRα1-mMOG-35-55 is a potent therapeutic agent to treat EAE in mouse strains expressing different MHC genes induced with a different peptide than that contained within the DRα1-mMOG-35-55 construct.

### DRα1-mMOG-35-55 treatment increases splenocyte number but does not alter their activation state

**Fig. 3:**
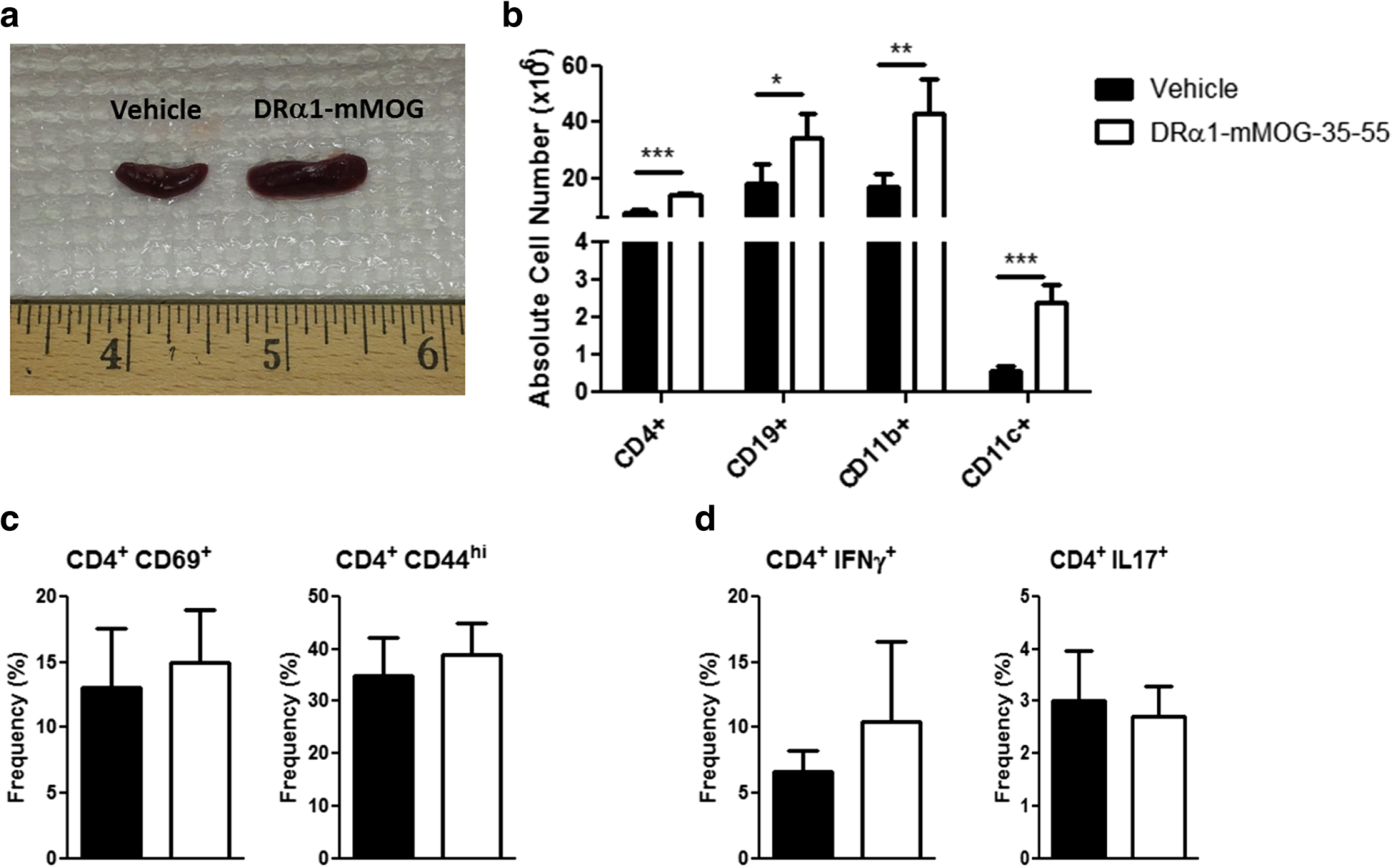
DRα1-mMOG-35-55 treatment increases splenocyte number but does not affect their activation state. **a** Representative image of spleens of vehicle-treated (*n* = 8) or DRα1-mMOG-35-55-treated (*n* = 8) C57BL/6 male WT mice with EAE. **b** Absolute cell numbers of CD4^+^, CD19^+^, CD11b^+^, and CD11c^+^ cells in spleen. **c** Frequency of CD4^+^CD69^+^ and CD4^+^CD44^+^ (activated CD4^+^ T cells) in spleen. **d** Splenocytes from DRα1-mMOG-35-55-treated (*n* = 4) and vehicle-treated (*n* = 4) mice were stimulated with PMA (50 ng/ml) and ionomycin (500 ng/ml) for 4 h. CD4^+^ T cells were evaluated for the intracellular expression of IFN-γ or IL-17 by flow cytometry. Data are presented as mean ± SD. *p < 0.05, **p < 0.01, ***p < 0.001, Student’s *t* test

To evaluate the effects of the DRα1-mMOG-35-55 construct on inflammatory cells in the periphery, spleen cells were isolated and analyzed on day 5 post-treatment (24 h after the last treatment). As shown in Fig. 3a, b, the absolute number of spleen cells was significantly increased in DRα1-mMOG-35-55-treated mice compared with vehicle-treated mice (148.5 × 10^6^ ± 37.9 vs. 64.7 × 10^6^ ± 19.0, respectively, *p* < 0.001) as were the absolute numbers of CD4^+^, CD19^+^, CD11b^+^, and CD11c^+^ spleen cell subtypes. However, analysis of expression of activation markers (CD44 and CD69) on CD4^+^ T cells revealed no significant differences in splenocytes from mice with EAE after DRα1-mMOG-35-55 vs. vehicle treatment. In addition, there was no difference in the intracellular expression of IFN-γ or IL-17 after PMA/ionomycin stimulation of spleen cells from mice that were treated with DRα1-mMOG-35-55 vs. vehicle (Fig. 3c, d).

### DRα1-mMOG-35-55 treatment reduces the number of CNS-infiltrating cells and their activation state

**Fig. 4:**
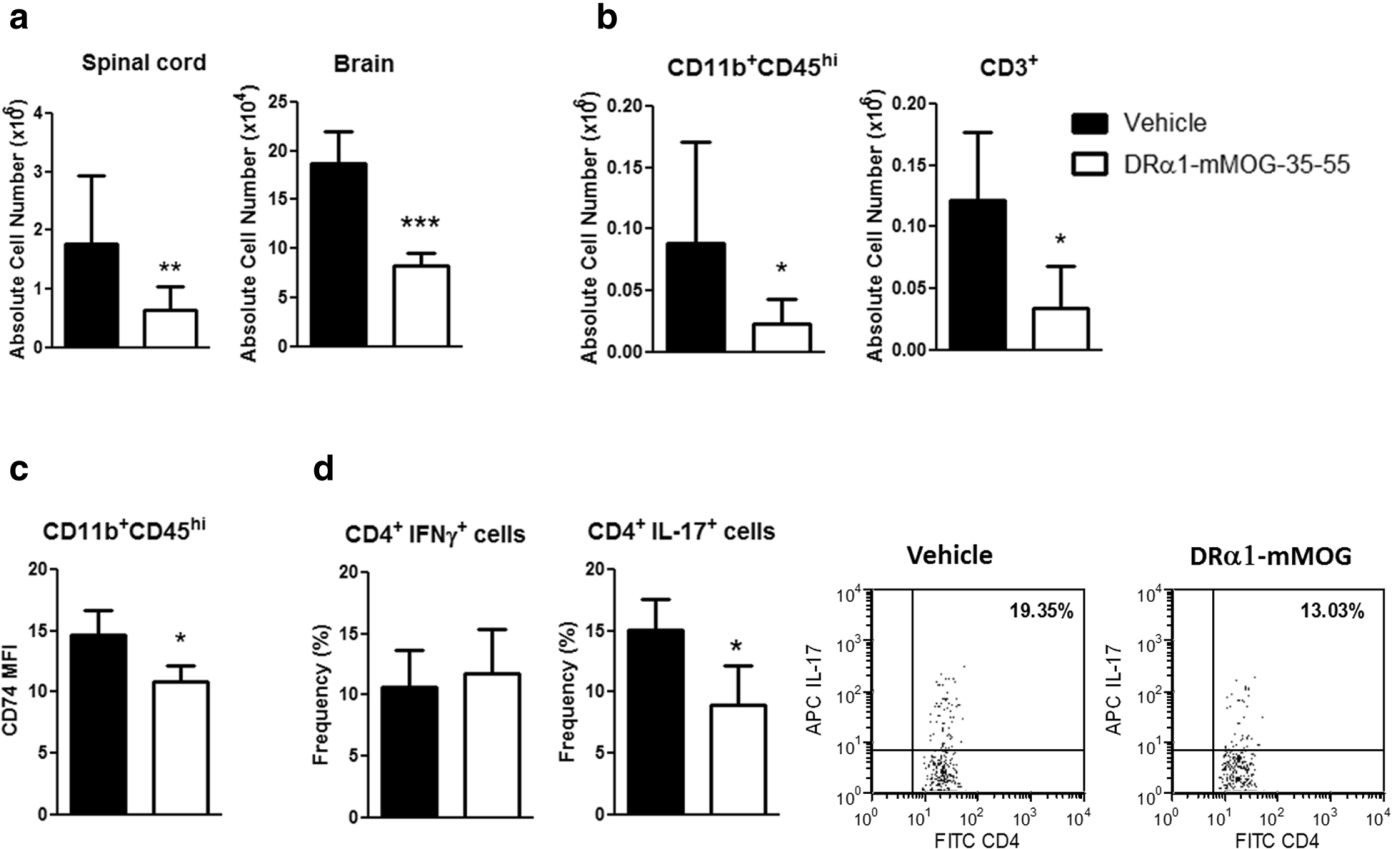
DRα1-mMOG-35-55 treatment reduces the number and activation state of CNS-infiltrating cells. **a** Absolute lymphocyte numbers in the spinal cord (*n* = 8) and brain (*n* = 4) from DRα1-MOG-35-55- or vehicle-treated C57BL/6 male WT mice with EAE. **b** Absolute numbers of CD11b^+^CD45^high^ and CD3^+^ T cells in spinal cords. **c** CD74 cell surface expression on CD11b^+^CD45^high^ cells in spinal cords. **d** Brain lymphocytes from DRα1-mMOG-35-55-treated (*n* = 4) and vehicle-treated (*n* = 4) mice were stimulated with PMA (50 ng/ml) and ionomycin (500 ng/ml) for 4 h. CD4^+^ T cells were evaluated for the intracellular expression of IFN-γ or IL-17 by flow cytometry. Data are presented as mean ± SD. *p < 0.05, **p < 0.01, ***p < 0.001, Student’s *t* test

We reported previously that two-domain partial MHC class II constructs could reduce EAE severity and CNS inflammation by reducing the number and frequency of activated cells in DR*1501-Tg mice. In order to evaluate if DRα1-mMOG-35-55 treatment of C57BL/6 mice had a similar effect, mononuclear cells were isolated from brains and spinal cords from DRα1-mMOG-35-55- or vehicle-treated mice 5 days post-EAE treatment. The absolute number of mononuclear cells was reduced both in the brain (8.25 × 10^4^ ± 1.2 vs. 18.75 × 10^4^ ± 3.2, *p* < 0.001) and spinal cord (0.64 × 10^6^ ± 0.4 vs. 1.76 × 10^6^ ± 1.15, *p* < 0.01), respectively, of DRα1-mMOG-35-55-treated vs. vehicle-treated mice with EAE (Fig. 4a). This reduction was reflected in the absolute number of infiltrating monocytes and activated resident microglia (CD11b^+^CD45^hi^) as well as CD3^+^ T cells in the spinal cord (*p* < 0.05, Fig. 4b). In addition, the CD74 expression level on CD11b^+^CD45^hi^ cells was significantly lower in the spinal cords of DRα1-mMOG-35-55- vs. vehicle-treated mice (*p* < 0.05, Fig. 4c), in accordance with effects of the parent DR*1501 β1α1-mMOG-35-55 construct in DR*1501-Tg mice [30]. Furthermore, there was a significantly lower frequency of PMA/ionomycin-stimulated IL-17^+^ T cells but not CD4^+^ IFN-γ^+^ Τ cells from DRα1-mMOG-35-55-treated mice compared with stimulated cells from vehicle-treated mice (*p* < 0.05, Fig. 4d).

Taken together, these data suggest that DRα1-mMOG-35-55 inhibits migration of activated inflammatory cells from the periphery to the CNS of C57BL/6 mice and that the infiltrating cells in the spinal cords of DRα1-mMOG-35-55-treated mice have reduced expression of CD74 and are less inflammatory compared with spinal cord cells from vehicle-treated mice.

### DRα1-mMOG-35-55 treatment enhances the frequency of CD11b^+^CD206^+^ M2 macrophages in spinal cords of mice with EAE

**Fig. 5:**
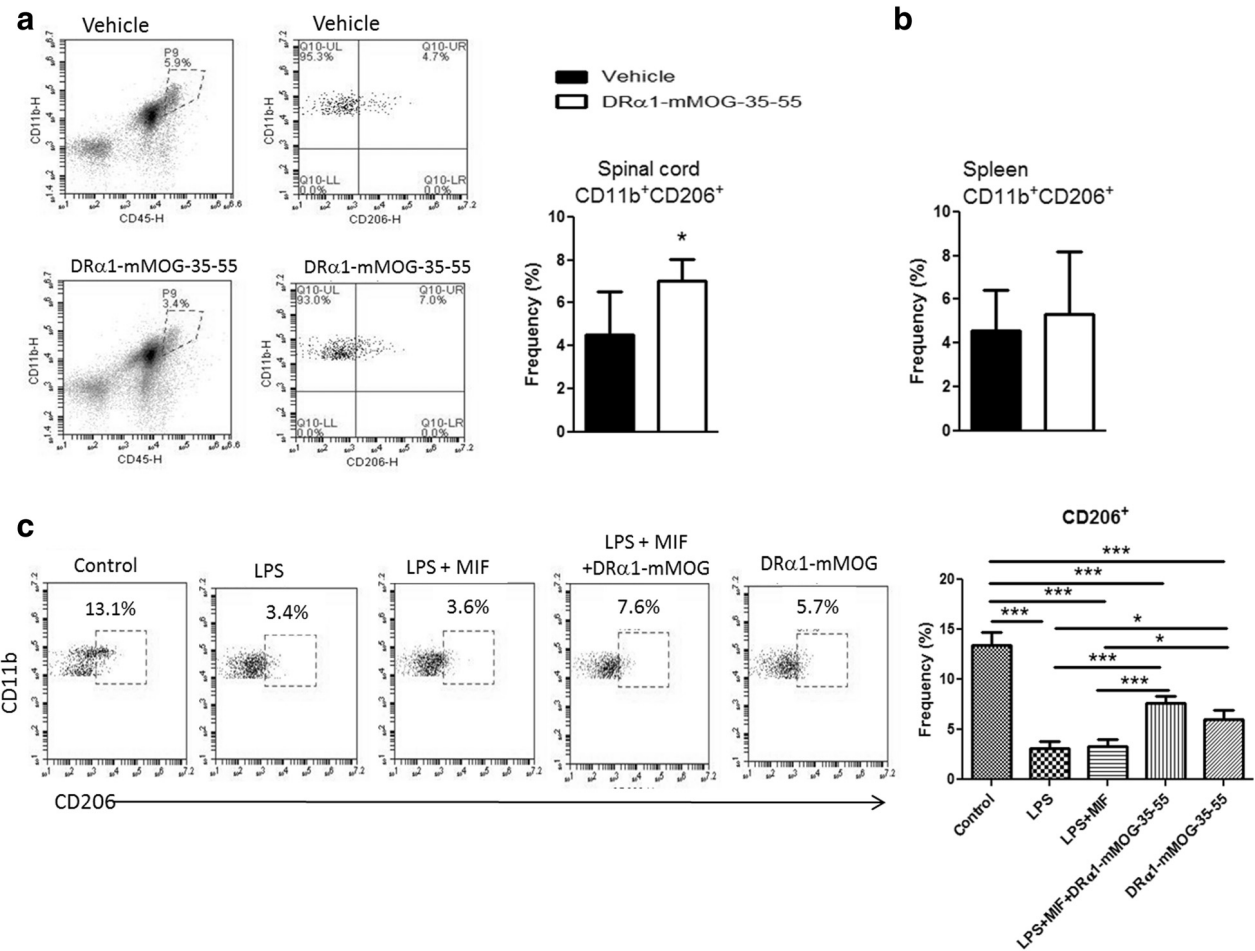
DRα1-mMOG-35-55 treatment increases the frequency of CD11b^+^CD206^+^ M2 macrophages in spinal cords of mice with EAE. **a** Frequency of CD206^+^ on CD11b^+^CD45^hi^ cells from spinal cords of DRα1-mMOG-35-55-treated (*n* = 6) vs. vehicle-treated (*n* = 6) C57BL/6 male WT mice with EAE. **b** Frequency of CD206^+^ on CD11b^+^CD45^hi^ cells from spleens of DRα1-mMOG-35-55-treated (*n* = 6) or vehicle-treated (*n* = 6) C57BL/6 male WT mice with EAE. **c** Splenocytes from C57BL/6 male WT mice (*n* = 4) with EAE (day 15 post-immunization) were treated with 10 ng/ml LPS, 100 ng/ml recombinant MIF, and 25 μg/ml DRα1-mMOG-35-55 for 24 h. Frequency of CD206^+^ on CD11b^+^F4/80^+^ cells was evaluated by flow cytometry. Data are presented as mean ± SD. *p < 0.05, **p < 0.01, ***p < 0.001. **a**, **b** Student’s *t* test. **c** One-way ANOVA with Tukey post-test

During EAE, M1 macrophages were shown to induce CNS inflammation, whereas M2 macrophages were shown to be involved in neuroprotection and remyelination [22, 24, 23]. We analyzed the frequency of activated CD11b^+^CD206^+^ M2 macrophages in spinal cords of C57BL/6 mice with EAE 24 h after the last treatment with DRα1-mMOG-35-55 or vehicle. As shown in Fig. 5a, the frequency of M2 macrophages (CD11b^+^CD206^+^) was significantly increased in DRα1-mMOG-35-55-treated vs. vehicle-treated mice (*p* < 0.05). In contrast, no difference in CD11b^+^CD206^+^ frequency was observed in the periphery (Fig. 5b).

In order to determine whether DRα1-mMOG-35-55 could directly induce M2 polarization, we isolated spleen cells from untreated C57BL/6 mice with EAE on day 15 post-immunization and stimulated them with LPS, LPS/MIF, LPS/MIF/DRα1-mMOG-35-55, or DRα1-mMOG-35-55 alone for 24 h at 37 °C. As expected, LPS or LPS/MIF treatment reduced the CD11b^+^CD206^+^ frequency (*p* < 0.001). However, treatment with LPS, MIF and DRα1-mMOG-35-55, or DRα1-mMOG-35-55 alone significantly increased the frequency of CD11b^+^CD206^+^ cells compared to LPS or LPS + MIF treatment (*p* < 0.001), but not to control levels (Fig. 5c).

We confirmed the increase in CD11b^+^CD206^+^ cells in spinal cords from DR*1501-Tg mice that were immunized with mMOG-35-55/CFA/Ptx and treated with DRα1-mMOG-35-55 (*p* < 0.05, Additional file 1: Figure S2a). Moreover, in vitro stimulation of spleen cells from naïve DR*1501-Tg mice showed similar results of an increased frequency of CD11b^+^CD206^+^ cells after DRα1-mMOG-35-55 treatment compared to LPS or LPS/MIF (*p* < 0.05, Additional file 1: Figure S2b), but not greater than controls. Thus, we conclude that DRα1-mMOG-35-55 treatment inhibits the pro-inflammatory effect of M1 polarization conditions but apparently does not directly induce M2 polarization.

### DRα1-mMOG-35-55 treatment of EAE reduces the expression of pro-inflammatory genes and increases the expression of genes involved in neurosurvival and regeneration

**Fig. 6:**
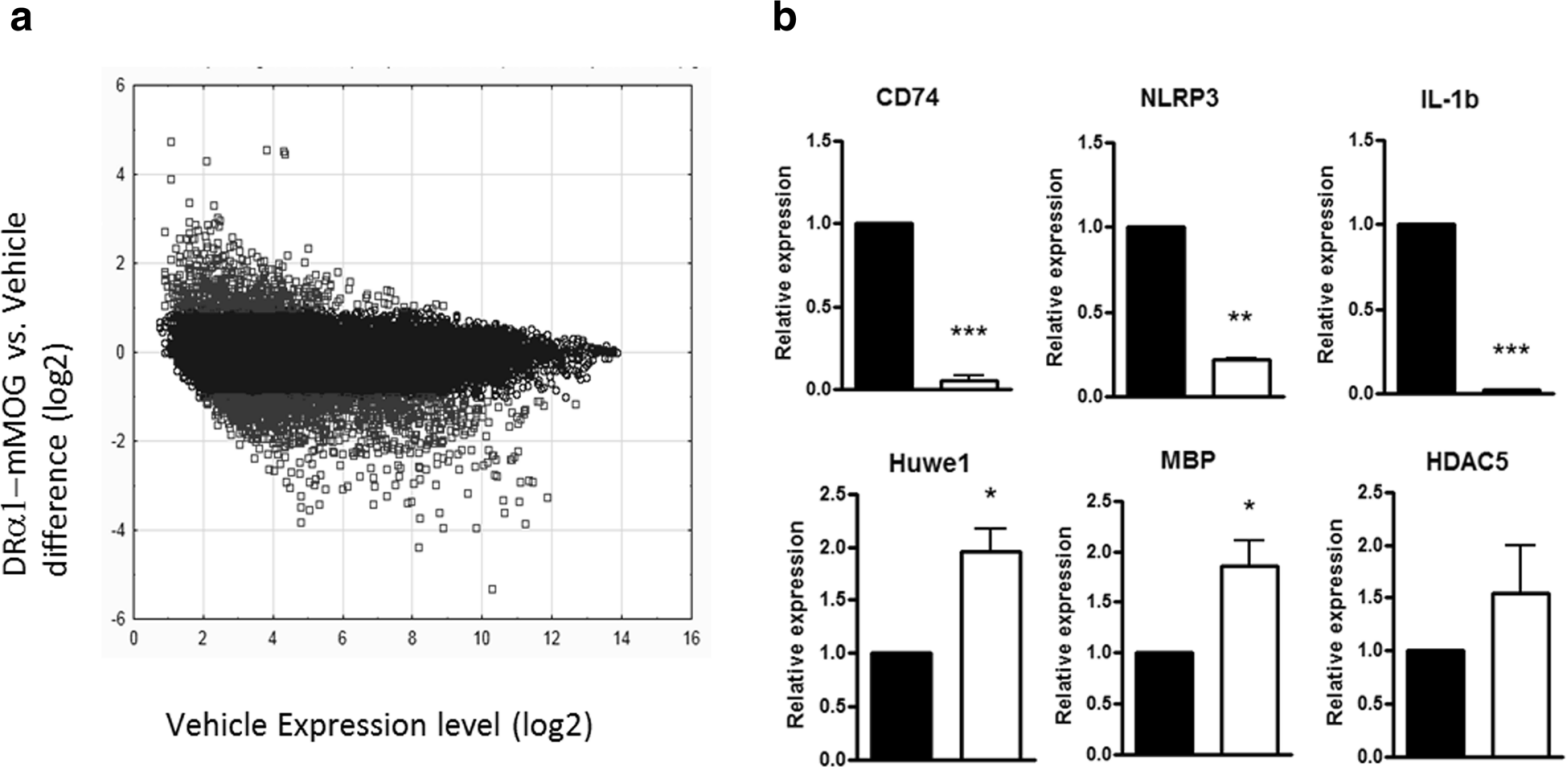
DRα1-mMOG-35-55 treatment reduces the expression of pro-inflammatory genes and increases the expression of genes involved in neurosurvival and regeneration. **a** Gene expression profile of spinal cords of DR*1501-Tg mice with EAE that were treated with DRα1-mMOG-35-55 (*n* = 3) or vehicle (*n* = 3). mRNA was pooled for microarray analysis. *Gray circles* represent probes that were at least twofold down- or upregulated after DRα1-mMOG-35-55 treatment relative to vehicle treatment. **b** Relative expression of mRNA of CD74, NLRP3, IL-1b, HUWE1, MBP, and HDAC5 was analyzed by real-time PCR from spinal cord samples of DRα1-MOG-35-55-treated (*n* = 3) relative to vehicle-treated (*n* = 3) mice. *p < 0.05, **p < 0.01, *p* < 0.001, Student’s *t* test

To evaluate DRα1-mMOG-35-55 effects on CNS inflammation during EAE in a more comprehensive manner, we performed microarray analysis on spinal cords from DRα1-mMOG-35-55- vs. vehicle-treated DR*1501-Tg mice with EAE. EAE was induced with mMOG-35-55/CFA/Ptx, and mice were treated with DRα1-mMOG-35-55 (100 μg daily × 3) or vehicle after disease onset at a clinical score of 2. Twenty-four hours after the last treatment, total RNA was isolated from spinal cords and gene expression profiles from pooled RNA were analyzed using the Mouse Gene 2.0 ST Affymetrix GeneChip system (Geo: microarray data GSE68805 and Additional file 2: Table S1). As shown in Fig. 6a, relative up- or downregulated genes after treatment with DRα1-mMOG-35-55 plotted against their expression level in spinal cords of vehicle-treated mice revealed 1049 probes that were downregulated by twofold or greater. Out of these probes, 160 genes were shown to be involved in inflammation processes. Conversely, 568 probes were upregulated by twofold or greater in the spinal cord of DRα1-mMOG-35-55-treated vs. vehicle-treated mice. Gene ontology analysis of the genes that were upregulated did not indicate a distinct pathway that was enriched as was observed for downregulated pro-inflammatory response genes. However, several of the genes that were relatively upregulated by DRα1-mMOG-35-55 treatment were shown to be involved in neuroregeneration, including Prosaposin (PSAP), Myocilin (MYOC), E3 ubiquitin ligase Huwe1, and 3-phosphoinositide-dependent protein kinase 1 (PDPK-1) [35–40]. We validated these microarray results by real-time PCR analysis of spinal cord mRNA from three individual mice in each group. Indeed, pro-inflammatory genes such as CD74, Nlrp3, and IL-1b were significantly downregulated in spinal cords of DRα1-mMOG-35-55-treated vs. vehicle-treated mice (*p* < 0.001, *p* < 0.01, and *p* < 0.001, respectively), whereas Huwe1 and MBP were significantly upregulated (*p* < 0.05). Histone deacetylase 5 (HDAC5) was also upregulated, although results did not reach significance (Fig. 6b) [41, 42]. These results indicate the CNS of DRα1-mMOG-35-55-treated mice is not only less inflammatory but also that this treatment could inhibit and potentially reverse ongoing demyelination and neurodegeneration.

## Discussion

Two-domain α1β1/peptide partial MHC class II constructs were shown to be highly beneficial in treating EAE, a widely utilized animal model for MS [43, 30]. One such single-exon construct, RTL1000, containing the human MOG-35-55 peptide linked to the DR2β1 and DRα1 domains through a flexible linker was recently tested successfully in a phase 1 clinical trial in HLA-DR2^+^ MS subjects [44]. However, treatment with RTL1000 would currently require HLA screening of ~50 % of MS patients who are DR2+, with the remaining 50 % of HLA-mismatched subjects not eligible to enroll in RTL1000 trials. A major goal of this study was to determine if the DRα1-mMOG-35-55 construct could effectively treat EAE in C57BL/6 mice, in which the DRα1 domain represents a mismatch with the mouse H-2 MHC molecules. The results of this study clearly demonstrate that DRα1-mMOG-35-55 treatment of EAE in C57BL/6 mice produced a highly significant reduction of CNS inflammation, mainly by inhibiting the infiltration of pro-inflammatory cells from the periphery. We recently demonstrated that the DRα1-mMOG-35-55 construct could treat experimental stroke in DR*1502-Tg mice. Four daily treatments with DRα1-mMOG-35-55 reduced infarct size by 40 % in the cortex, striatum, and hemisphere; inhibited the migration of activated CD11b^+^CD45^high^ cells from the periphery to the brain; and reversed splenic atrophy [45]. In the current study, we also show that DRα1-mMOG-35-55 can effectively treat DR*1502-Tg mice with EAE induced by human MOG-35-55 peptide, a different model with minor MHC and peptide mismatches with the DRα1-mMOG-35-55 construct. DRB1*1502 is nearly identical to DRB1*1501 in primary amino acid sequence except for a glycine for valine substitution at position 86 that contributes to the P1 pocket involved in peptide binding. The smaller glycine residue at position 86 of the DRB1*1502 molecule likely enlarges the P1 pocket to permit binding of large hydrophobic residues such as tyrosine or tryptophan such as that found in m or hMOG-35-55, which are not bound as easily by the P1 pocket in the DRB1*1501 allele.

In the DR*1502-Tg mice, the DRB1*1502 transgene pairs with the mouse I-Eα^k^ chain to form a functional MHC complex on the cell surface [46]. I-A is not expressed in these mice because they were derived from Ab0 mouse class II knockout mice [47]. Responses to antigen in these mice are thus restricted mainly by human DR2, and to that extent, they are able to pair in mice expressing the DRB1*1502 transgene, possibly by mouse class II I-Eα^k^/I-Eβ^b^.

In addition, we previously found that DRα1-mMOG-35-55 could attenuate MBP-induced EAE [29]. Hence, this novel construct could treat different CNS diseases in different mouse strains that are mismatched in the DRα1 domain or diseases that were induced by a different myelin peptide than mMOG-35-55 that is covalently linked to the DRα1 domain. Although not fully addressed in this study, these inhibitory effects may involve MIF blockade by antagonist interactions between DRα1-mMOG-35-55 and mouse CD74 consistent with our previous observations in DR*1501-Tg mice derived on the C57BL/6 background.

Previously, we showed that the DRα1 domain binds to CD74 on the monocyte cell surface and downregulates CD74 expression and blocks MIF binding and signaling [48, 7]. This blockade of MIF signaling affects cell migration into the CNS during EAE, thus resulting in a less inflammatory milieu. Indeed, treatment with the DRα1-mMOG-35-55 construct reduced expression of CD74, resulting in a significant decrease in the absolute number of activated CD11b^+^ macrophages/monocytes and T cells in the CNS of C57BL/6 mice. It is important to note that this effect was due not only to blocking the entry of infiltrating cells into the CNS but also to previously infiltrated cells since mice were treated with DRα1-mMOG-35-55 at the onset of marked clinical signs, well after the entry of infiltrating cells into the CNS.

Our data indicate that the DRα1-mMOG-35-55 construct, which contains the human HLA-DRα1 domain, could effectively bind to the mouse CD74 molecule. This might be due to its high level of similarity (~75%) with the human CD74 molecule. We are currently trying to identify the exact binding region of the DRα1 on CD74. Furthermore, although we previously showed that CD11b^+^ monocytes are the predominant cell population that is binding the DRα1 constructs, it is clear that in vivo treatment affects other cell types as well. We demonstrated that CD4^+^ T cells in the CNS of DRα1-mMOG-35-55-treated mice express lower levels of IL-17 compared with vehicle-treated mice. Whether this effect could be direct or indirect is the subject of a current investigation.

Macrophages have a central role in MS pathology [20, 26, 22, 49]. They are involved in different stages of the disease and assume a diversity of distinct activation states. Although the range of activation of macrophages is very wide, it is acceptable to characterize at least two opposing activation states: The classically activated macrophages (M1) express high levels of CD86, CD80, and MHC class II on their cell surface and are very potent in priming T cells and recruiting them to the CNS. These cells are predominantly present in the early stages of EAE. One the other side of the activation spectrum, alternatively activated macrophages (M2) express high levels of CD206, CD163, and arginase1 and low levels of CD40, CD86, and MHC class II [21]. Several studies suggested that M2 macrophages have a beneficial function in EAE, both in inhibiting inflammation and in inducing remyelination by phagocytosis of myelin debris [24, 25, 28]. In our present work, we found an increased frequency of CD206^+^ CD11b^+^CD45^hi^ cells in the spinal cords of DRα1-mMOG-35-55- vs. vehicle-treated mice with EAE. This was observed both in C57BL/6 and in DR*1501-Tg mice. This observation is in line with the low expression levels of CD74 on these activated cells from treated mice. Interestingly, there was no difference in the frequency of CD206^+^ monocytes in the periphery. Furthermore, in vitro analysis of activated cells showed that while LPS or LPS and MIF stimulation decreased the frequency of CD206^+^CD11b^+^ cells, DRα1-mMOG-35-55 treatment partially reversed the LPS effect. However, treatment with DRα1-mMOG-35-55 alone did not induce a higher frequency of CD11b^+^CD206^+^ cells above the control level, suggesting that DRα1-mMOG-35-55 has an inhibitory effect on pro-inflammatory stimuli rather than a direct stimulatory effect on M2 cells. It was reported previously that MIF-KO mice have a higher frequency of M2 cells compared with WT mice [50]. In addition, since we previously demonstrated that DRα1 blocks MIF binding, spleen cells were stimulated in vitro with LPS or LPS and MIF in order to evaluate that effect of MIF on M2 reduction and the role of DRα1-mMOG-35-55 in blocking MIF signaling. However, we did not detect an additive effect of the MIF over the LPS. It is possible that effects of added exogenous MIF are not detectable since LPS stimulation is inducing sufficient endogenous MIF. It would be of interest in future studies to assess the M1/M2 ratio in the CNS of MIF-deficient mice with EAE.

Analysis of a gene expression profile of spinal cords from DRα1-mMOG-35-55-treated mice relative to vehicle-treated mice demonstrated a wide reduction in the expression of pro-inflammatory genes. This effect might be due to the combination of a low number of activated cells in the spinal cord of DRα1-mMOG-35-55-treated mice and to the DRα1-mMOG-35-55 effect on the activation levels of the inflammatory cells that are in the CNS.

We also demonstrated for the first time that the DRα1-mMOG-35-55 construct could increase the expression of genes that are involved in myelin repair and neural survival or regeneration relative to vehicle. In addition to MBP expression that was elevated in the DRα1-mMOG-35-55-treated group, we observed increased expression of genes such as HUWE1, PROSAPOSIN, and MYOCILIN. The ubiquitin ligase HUWE1 was shown to regulate glia differentiation and to have an essential role in the proliferation and differentiation of neural progenitor cells [35, 39]. PROSAPOSIN is the precursor protein for four lysosomal activator proteins (saposins A–D), and it can also be secreted as a full-length protein. It was shown that PROSAPOSIN could protect myelinating glial cells, enhance nerve regeneration, and promote synaptic development [38]. It was recently shown that MYOCILIN is involved in myelination of the optic nerve in mice [37]. It is possible that during the acute phase of EAE, the expression levels of these genes are downregulated in the CNS compared with a less inflammatory CNS. Interestingly, in a previous work, it was shown that treatment of EAE with a partial MHC class II construct could reduce demyelination, axonal loss, and ongoing damage [51]. Those experiments also suggested that such treatment could induce repair of myelin and axonal damage. Additional experiments are in progress to determine whether DRα1-mMOG-35-55 can directly induce repair of axonal damage during EAE.

## Conclusion

In summary, our work indicates that the DRα1-mMOG-35-55 construct could treat EAE in part by reducing CNS inflammation. Because the DRα1 and the CD74 (MHC class II invariant chain) amino acid sequences are conserved in humans, the recombinant DRα1-MOG-35-55 construct potentially represents an immunotherapy that would not require HLA screening prior to use. In addition to inhibition of pro-inflammatory factors, we show an increase in the frequency of M2 macrophages in the spinal cord of DRα1-mMOG-35-55-treated mice. Thus, our data demonstrate that this treatment could inhibit additional damage caused by inflammation and set the appropriate conditions for repair of myelin and axonal damage.

## Additional files

Additional file 1:
**Supplementary figures. Figure S1**. DRα1-mMOG-35-55 treats clinical EAE in DR*1502-Tg mice. **Figure S2**. DRα1-mMOG-35-55 treatment increases the frequency of CD11b^+^CD206^+^ in spinal cords of DR*1501-Tg mice with EAE. Additional file 2:
**Gene expression profiles. Table S1**. Spinal cord gene expression profiles from pooled RNA analyzed using the Mouse Gene 2.0 ST Affymetrix GeneChip system.
