# Supplementary material for: HLA-DRα1-mMOG-35-55 treatment of experimental autoimmune encephalomyelitis reduces CNS inflammation, enhances M2 macrophage frequency, and promotes neuroprotection
Source: J Neuroinflammation. 2015 Jun 24;12:123. doi: 10.1186/s12974-015-0342-4 (PMC4481122; doi:10.1186/s12974-015-0342-4)
Supplement: Additional file 1: — Supplementary figures. Figure S1. DRα1-mMOG-35-55 treats clinical EAE in DR*1502-Tg mice. Figure S2. DRα1-mMOG-35-55 treatment increases the frequency of CD11b+CD206+ in spinal cords of DR*1501-Tg mice with EAE. [file 12974_2015_342_MOESM1_ESM.pdf]

Supplementary figures.

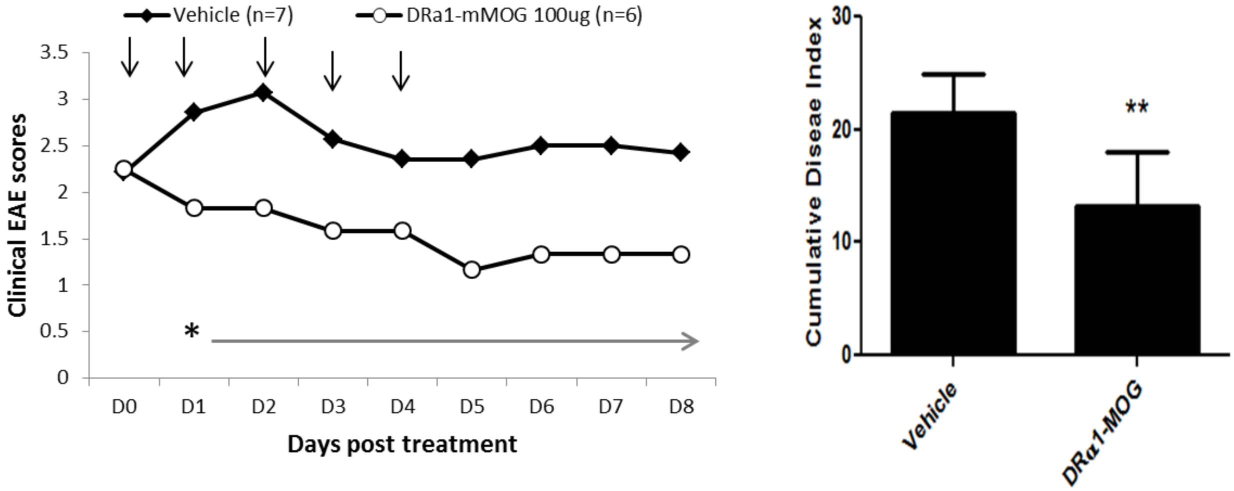

Supplementary Figure 1. DRα1−mMOG-35-55 treats clinical EAE in DR*1502-Tg mice. DR*1502-Tg mice with human MOG-35-55-induced EAE were treated after disease onset at a clinical score of ≥2 with DRα1−mMOG-35-55 (100 μg, daily x 5, black arrows) or Vehicle. Mean clinical EAE daily disease scores (left) and cumulative disease index scores (right) are shown. *p<0.05, **p<0.01. Daily mean scores were analyzed by Mann Whitney U and mean CDI by Student's t-test.

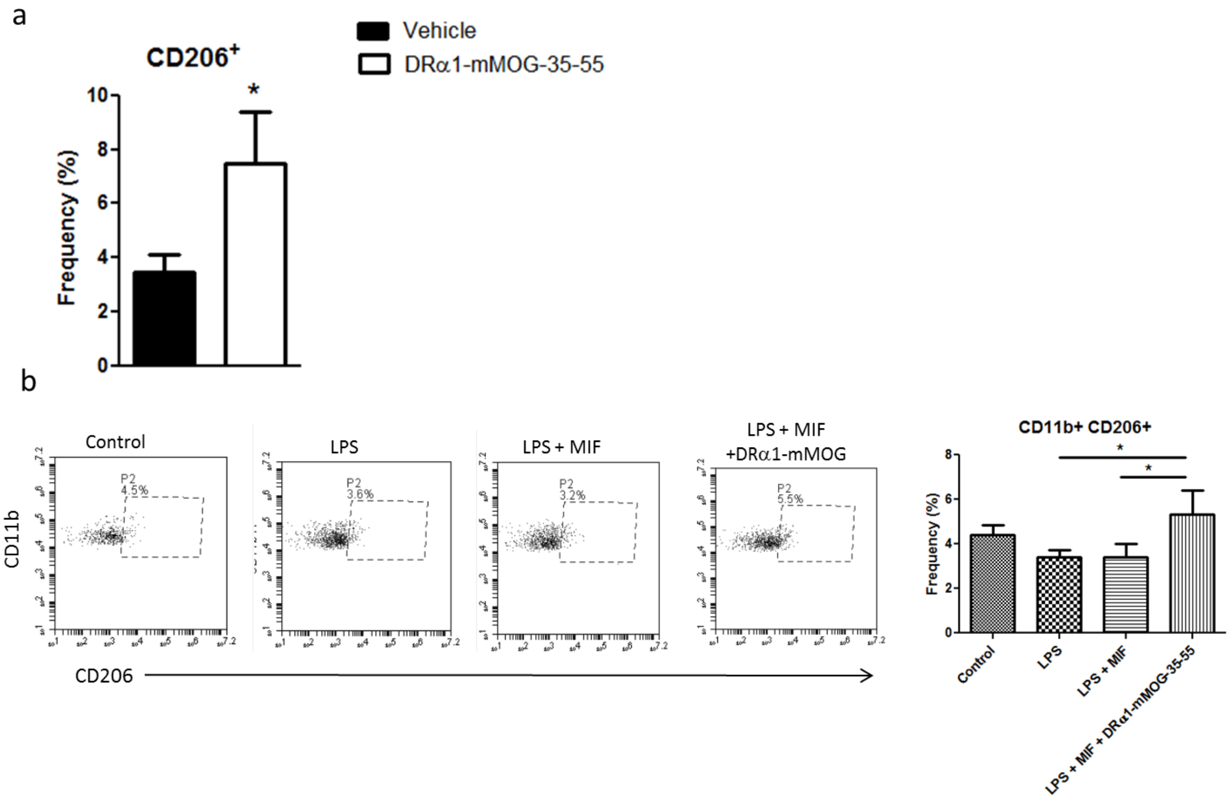

Supplementary Figure 2. DRα1-mMOG-35-55 treatment increases the frequency of CD11b$^+$CD206$^+$ in spinal cords of EAE mice. (a) Frequency of CD206$^+$ on CD11b$^+$CD45^{hi} cells from spinal cords of DRα1-mMOG-35-55- (n=6) vs. Vehicle-treated (n=6) DR*1501-Tg mice with EAE. (b) Splenocytes from naïve DR*1501-Tg mice (n=4) were treated with 10ng/ml LPS, 100ng/ml recombinant MIF and 25μg/ml DRα1-mMOG-35-55 for 24 hr. Frequency of CD206$^+$ on CD11b$^+$F4/80$^+$ cells was evaluated by flow cytometry. Data are presented as mean ± SD. * $p<0.05$, (a) Student's t-test. (b) One way ANOVA with Tukey post-test.
